# Milk intake enhances cerebral antioxidant (glutathione) concentration in older adults: A randomized controlled intervention study

**DOI:** 10.3389/fnut.2022.811650

**Published:** 2022-08-15

**Authors:** In-Young Choi, Matthew K. Taylor, Phil Lee, Sibelle Alwatchi Alhayek, Misty Bechtel, Jill Hamilton-Reeves, Kendra Spaeth, Peter Adany, Debra K. Sullivan

**Affiliations:** ^1^Department of Neurology, University of Kansas Medical Center, Kansas City, KS, United States; ^2^Hoglund Biomedical Imaging Center, University of Kansas Medical Center, Kansas City, KS, United States; ^3^Department of Radiology, University of Kansas Medical Center, Kansas City, KS, United States; ^4^Department of Molecular and Integrative Physiology, University of Kansas Medical Center, Kansas City, KS, United States; ^5^Department of Dietetics and Nutrition, University of Kansas Medical Center, Kansas City, KS, United States; ^6^Department of Urology, University of Kansas Medical Center, Kansas City, KS, United States

**Keywords:** brain glutathione (GSH), milk intervention, older adults, randomized controlled clinical trial (RCT), aging, magnetic resonance spectroscopy (MRS), reactive oxygen species (ROS), oxidative stress

## Abstract

**Background:**

A major antioxidant, glutathione (GSH), is a key factor in the antioxidant defense mechanism against oxidative stress and aging-related functional declines. Our previous observational study showed positive correlations between brain GSH concentrations and dairy food consumption, particularly milk (*p* < 0.001), in older adults.

**Objective:**

To investigate whether a recommended amount of milk intake (3 cups/day) in low dairy consumers enhances brain GSH concentrations through an intervention trial.

**Methods:**

Seventy-three older adults (60–89 years) with a low dairy intake (≤1.5 servings/day) were randomized (5:2 ratio) in this 3-month randomized clinical trial. The intervention group was provided 1% milk weekly and instructed to consume 3 cups of milk/day for 3 months while the control group continued their habitual intake of total dairy ≤ 1.5 servings/day (<1 cup of milk/day). Brain GSH concentrations were measured in the fronto-parietal region using our unique 3 T magnetic resonance chemical shift imaging technique at baseline and 3 months.

**Results:**

Among 73 randomized participants, 66 participants (49 intervention; 17 controls) completed the study. Milk intake in the intervention group increased from 0.2 ± 0.3 cups/day to 3.0 ± 0.6 cups/day (*p* < 0.001) between baseline and the end of the study, while milk intake in the control group did not differ throughout the study duration (0.4 ± 0.4 cups/day). The intervention group showed increases in brain GSH concentrations by 7.4 ± 11.7% (*p* < 0.001) in parietal and 4.7 ± 9.8% (*p* = 0.003) in fronto-parietal regions, and 4.6 ± 8.7% (*p* < 0.001) in overall brain concentration after the intervention compared with baseline, while the control group showed no changes.

**Conclusion:**

This study provides evidence that milk serves as a good dietary source to increase and/or restore brain GSH concentrations in older adults. Identifying dietary sources that effectively enhance antioxidant defenses and neuroprotection could lead to the development of new strategies to promote brain health in the aging population.

**Clinical trial registration:**

[https://ClinicalTrials.gov], identifier [NCT02957422].

## Introduction

Our previous finding (1) that dairy consumption and brain glutathione (GSH) concentrations were positively correlated in older adults prompted public interest in the dietary intake of foods (e.g., milk) that could promote brain health, *via* enhancing cerebral antioxidant defenses against oxidative stress, a major mechanism of aging and neurodegeneration (2–5). Antioxidant defenses rely on the function of antioxidants in protecting cells from excessive reactive oxygen species (ROS) and free radicals, which lead to oxidative stress-induced cellular damage, impaired cell function, and eventual cell death. The nature of the brain with a high metabolic rate of oxygen consumption makes the brain more prone to oxidative damage due to the high production of ROS in the mitochondria (6). Furthermore, the brain is highly sensitive to alterations in antioxidant defense systems. Therefore, antioxidant status is particularly important for the brain.

Glutathione is the most concentrated non-enzymatic free radical-scavenging antioxidant (7) and the primary regulator of cellular redox state and functions in the brain (8). GSH provides the first line of defense against ROS, especially singlet oxygen and hydroxyl radicals. Currently, no other known enzymatic defenses exist to prevent or ameliorate the damaging effects of these ROS, except *via* the functions of GSH in the cerebral antioxidant defense system (9–11). Consequently, GSH concentrations are lower in brain tissue undergoing increased oxidative stress that occurs in aging and many neurodegenerative diseases (12, 13). In addition, loss of brain GSH has been linked to neuronal death in various neurological disorders (14). Thus, restoring or enhancing brain GSH concentrations could be a key factor in strengthening cerebral antioxidant defenses in the aging brain and may even provide therapeutic benefits for neurological diseases (15).

Our unique, non-invasive multiple quantum chemical shift imaging (CSI) technique allows direct measurements of brain GSH concentrations in the living human brain as we have described previously (16–19). Using this quantitative magnetic resonance (MR) technique of GSH CSI, our previous study suggested the possibility that the dietary intake of milk could lead to increases in brain GSH (1), possibly by providing adequate substrates that support GSH synthesis and maintenance in the aging brain. However, the cross-sectional study design precluded providing direct evidence of the effect of dairy consumption on brain GSH concentrations because of the limited capability of controlling various potential factors that could influence brain GSH concentration. Increases in brain GSH cannot be achieved by administration of GSH itself but through biosynthesis from its three substrate amino acids (cysteine, glutamate, and glycine) in the brain. All three of these amino acid substrates are contained in milk. The availability of cysteine within the cell is a significant rate-limiting factor in GSH synthesis, thus its sufficient supply relies on dietary intake (20–22). Furthermore, milk is rich in riboflavin and calcium with high bioavailability (23), which are required for the maintenance of cellular GSH. Riboflavin is a cofactor of glutathione reductase necessary for the enzymatic reaction that converts glutathione disulfide (GSSG) to GSH (15). Therefore, here we designed a rigorous, randomized controlled trial of an intervention with milk to overcome the limitation of the cross-sectional study.

We hypothesize that milk supplies critical nutrients to support GSH synthesis (1). Therefore, in this study, we tested whether increasing milk intake to the recommended 3 cups/day in older adults with habitual low dairy consumption increases brain GSH concentrations.

## Materials and methods

### Study design

The milk intervention for longevity at the University of Kansas (MILK) study was a single-blind randomized clinical trial that tested the effect of a 3-month milk intervention (3 cups/day) on brain GSH concentrations in cognitively normal older adults with habitual low dairy intake. The intervention interval of 3 months was chosen based on our previous study demonstrating that a complete turnover of the brain GSH pool in the brains of rats takes approximately 3 days (24). Currently, no data are available on brain GSH metabolism and turnover for humans. Thus, given the slower metabolic rate for humans compared with rats and the estimated turnover rate and the time constant of GSH, we estimated that 3 months should be sufficient to reach steady-state metabolic conditions and observe dietary intervention-related changes in brain GSH concentrations.

The study design was informed by our previous observational study (1). A comparison of cross-sectional differences in fronto-parietal GSH concentration between low and recommended dairy consumers had an estimated Cohen’s d effect size of 1.28. Based on a more conservative effect size observed between the low and moderate milk consumption groups (Cohen’s d = 0.63) and an assumed α = 0.05, we estimated that a sample size of *n* = 22 in the intervention group would provide >80% power to detect within-group change in fronto-parietal GSH concentration. Based on these assumptions, we randomized *n* = 73 older adults with low dairy consumption in a 5:2 ratio to either the 3 cups/day milk intervention or the control group where participants were instructed to continue their regular dairy consumption habits. The unbalanced study design was intended to provide optimal power to detect changes in brain GSH concentrations within the intervention group and attain a comparative control group. The study was approved by the Institutional Review Board at the University of Kansas Medical Center (KUMC, Kansas City, KS, United States) and registered at www.ClinicalTrials.gov as NCT02957422. All data were collected and analyzed by research personnel blinded to participants’ group assignments except those involved in the intervention.

### Participants

**FIGURE 1:**
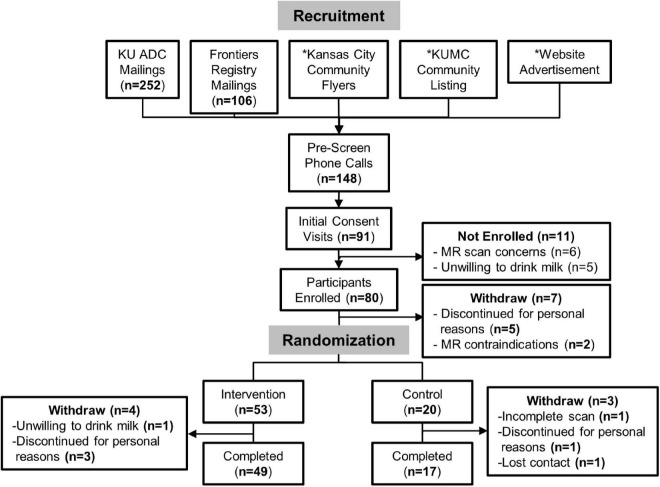
CONSORT diagram for MILK study recruitment and analysis. *We are unable to assess the number of individuals that came into contact with flyers and online study advertisements.

Participants were recruited from April 2017 to September 2019 from the Greater Kansas City area *via* printed flyers, research opportunity advertisements to the community, social media advertisements, and targeted registry mailings (Figure 1). Interested individuals first participated in an initial pre-screening telephone call to screen for key inclusion/exclusion criteria including the age of 60–89 years, being in good general health with no concomitant disease, a willingness to participate in a clinical trial, having no MRI contraindications, self-report of consuming an average of <1 cup of milk/day, and confirmed consumption of ≤1.5 servings/day of dairy foods assessed by one standardized, multiple-pass 24-h dietary recall. Participants that met pre-screen criteria were invited to attend the in-person screening visit where informed consent and full screening for eligibility were attained. In addition to the pre-screening requirements, participants were eligible if they had good general health attained by a full medical history, a Mini-Mental State Examination (MMSE) score ≥ 24, body mass index (BMI) between 18.5 and 35 kg/m^2^, and ≤1.5 servings/day of dairy as assessed by a second standardized 24-h dietary recall and the National Cancer Institute Dietary Screener Questionnaire (DSQ).

Our exclusion criteria included the presence of medical contraindications such as central neurological diseases, prior major head trauma with loss of consciousness, unstable and life-threatening systemic illness (e.g., cancer), diabetes mellitus (clinical diagnosis or use of an anti-diabetic agent), metabolic syndrome or uncontrolled hypertension, major psychiatric disorders within the past 3 years, depression (Geriatric Depression Score-Short Form > 5), and alcohol (>3 drinks/day or 18 drinks/week) or drug abuse. Participants were also excluded based on psychoactive drug or investigational medication use, supplements that might affect brain GSH (e.g., N-acetylcysteine), pregnancy, special diet regimens (e.g., vegan), and MR contraindications including devices, foreign objects, or claustrophobia. All subjects were asked to fast overnight before their study visits the following morning for serum cysteine measurements and MR scans at baseline and 3 months (Month 3). Eligible participants who reported consuming multivitamin or mineral supplements were asked to discontinue supplements and completed a 2-week washout period before attending the baseline study visit. We determined the duration of the washout period based on the estimated complete turnover time of GSH synthesis in the brain (25). All participants provided written informed consent to participate in the study per institutional guidelines.

### Randomization

**FIGURE 2:**
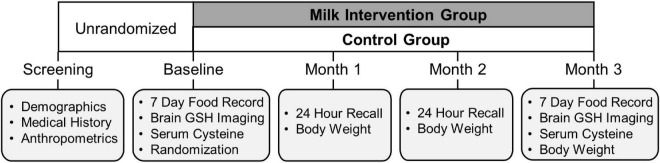
Timeline of the data collection of in-person MILK study visits.

The study randomization and intervention timeline are illustrated in Figure 2. After completion of the baseline MR scan, participants were randomly assigned in a ratio of 5:2 to either the intervention or control group using a computer-generated random-sequence allocation program. MR scan assessors were blinded to participant group allocation. After randomization, participants completed monthly mid-study visits at 1-month (Month 1), 2-month (Month 2), and the end of study visit at 3 months (Month 3) for the final measurement of outcomes of interest, including the final MR scan.

### The intervention group

The milk intervention group was provided conventional 1% milk weekly and instructed to consume 3 cups (237 mL/cup) of milk/day for 3 months. Low-fat 1% milk was chosen based on the Dietary Approaches to Stop Hypertension (DASH) diet recommendations (26, 27). We provided all participants with a 3-cup capacity shaker cup that included measurement indicators for each fluid ounce. A registered dietitian gave verbal and written instructions for participants to fill the container with 3-cups of milk and consume its entirety each day. To track intervention compliance, participants completed a daily milk consumption log indicating the number of unconsumed ounces of milk remaining in their 3-cup container daily. Furthermore, registered dietitians performed weekly 24-h dietary recalls by telephone for participants to recount their dairy intake on the previous day. We provided participants with 1.5 gallons/week of 1% milk from regional grocery store chains in the Kansas City metropolitan area. Participants had the option to choose weekly home delivery or pickup from the grocery store on a designated day and time each week. Confirmation of successful delivery or pickup was documented using an online portal accessible by study personnel. If special milk delivery arrangements were required, study personnel hand-delivered milk to participants at their homes.

### The control group

The control group was instructed to continue their habitual dietary intake, including an intake of ≤1.5 servings/day of dairy (<1 cup of milk/day). Both the control and intervention groups participated in the same study visits and outcomes measures at each visit.

### Anthropometric measures

Bodyweight, height, and waist circumference were measured at the eligibility screening visit. Bodyweight was measured with a calibrated digital scale (±0.1 kg; Befour, Saukville, WI, United States). Height was measured with a wall-mounted stadiometer (Model PE-WM-60-84; Perspective Enterprises, Portage, MI, United States). Waist circumference was measured in the standing position with measurements obtained midway between the lateral lower rib margin and the iliac crest (28). BMI was calculated using weight and height measurements. Bodyweight and height measurements were repeated monthly at Months 1, 2, and 3. Waist circumference measurement was repeated at the end of the study (Month 3).

### Dietary intake assessment

Dairy intake during the screening was assessed using two multiple-pass 24-h dietary recalls and the DSQ. The first 24-h recall was collected during the pre-screening telephone call to assess preliminary participant eligibility. The second 24-h recall was collected at the screening study visit. Dietary recalls were entered into the Nutrition Database System for Research (NDSR, version 2018) to quantify recent dietary intake on random days, including milk and total dairy. Participants also completed the DSQ at the screening visit. The DSQ is a 26-item questionnaire that assessed how frequently participants consumed several foods and beverages over the past month (29), providing an estimate of habitual milk and total dairy intake in real-time. The 24-h recall and DSQ assessments were used in tandem to ensure participants were eligible for study participation by reporting habitual consumption of ≤1.5 servings of dairy/day.

To assess dietary intake before MR scans at baseline and Month 3, 7-day food records (7DFR) were obtained from all participants. Participants began their 7DFR one week before the MR scan date with the final day of dietary intake collection ending the evening before their fasted MR scan visit the following morning. A study registered dietitian gave all participants both written and verbal instructions on accurately recording their dietary intake. On the day of the MR scan, the study registered dietitian reviewed the 7DFR with each participant for thoroughness and clarification. Dietary intake data was entered into NDSR to quantify food and nutrient intake.

We calculated diet quality scores for each participant using the Healthy Eating Index (HEI) 2015. The HEI-2015 is a measure of diet quality developed by the United States Department of Agriculture that assesses conformance to the 2015 Dietary Guidelines for Americans.^1^ The purpose of the HEI was to ensure that diet quality did not change within or between groups from the baseline to the end of study timepoints.

### Serum cysteine

Fasting blood draws were performed right before MR scans at baseline and Month 3 visits. Venous blood samples were collected into serum separator tubes and immediately centrifuged. Serum was taken off, aliquoted, and frozen at −80°C for later analysis. Total serum cysteine was quantified using fluorometric cysteine kinetic assay kits (BioVision Incorporated, California, United States).

### Magnetic resonance scan protocol

**FIGURE 3:**
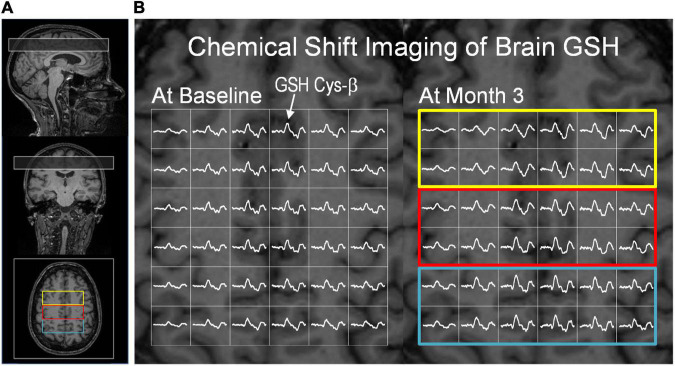
Glutathione CSI and creatine CSI data from a subject in the intervention group. **(A)** The position of the GSH CSI slab overlaid on T1-weighted MR images. The CSI slab indicated in a rectangle was placed superior to the corpus callosum in the fronto-parietal region of the brain. **(B)** Partial views of brain GSH CSI of a subject in the intervention group measured at baseline (left) and after the 3-month dietary intervention (right). Brain GSH CSI is overlaid on transverse T1-weighed MRI corresponding to the middle slice of the GSH CSI slab. Regions of interest are marked by colored rectangular boxes: (yellow: frontal; red: fronto-parietal; blue: parietal). The GSH signal from cysteinyl moiety at ∼3 ppm was marked by an arrow. Clear spectral patterns of GSH signals across the CSI slab indicate robustness and consistency of GSH measurements at baseline and post-intervention. The GSH spectra are shown in the chemical shift range of 2.6 – 3.4 ppm.

Magnetic resonance scans were performed at baseline and the end of study (Month 3) visits on a 3 T MR system (Skyra, Siemens, Erlangen, Germany) using a 20 channel receive RF coil. After positioning the subject supine in the magnet, three-plane scout MR images were acquired to locate the volume of interest (VOI), a 2.5 cm axial slab for chemical shift imaging (CSI) of brain GSH. The CSI slab was positioned above the corpus callosum parallel to the base of the corpus callosum (Figure 3) using an automatic slice positioning program. *In vivo* mapping of brain GSH was performed with a doubly selective multiple quantum filtered CSI technique (TE/TR = 115/1,500 ms, slice thickness = 2.5 cm, Field Of View (FOV) = 20 cm, matrix size = 10 × 10, scan time = 10 mins), utilizing a two-echo scheme that provides simultaneous measurements of GSH and creatine signals (17, 30, 31). Brain GSH scan was divided into four measurement blocks (2.5 mins of scan time/block) to minimize the effect of potential frequency changes due to static magnetic field instabilities and/or subject motion. Unsuppressed water CSI was also acquired from the same slab for an internal concentration reference and multi-channel signal combination. Three-dimensional T1-weighted MRI was acquired using an MPRAGE sequence (TE/TR/TI = 4.4/2,500/1,100 ms, resolution = 1 mm^3^ isotropic, matrix size = 192 × 192 × 176, GRAPPA factor = 2) for brain tissue partial volume correction and post-processing of brain GSH CSI data.

**FIGURE 4:**
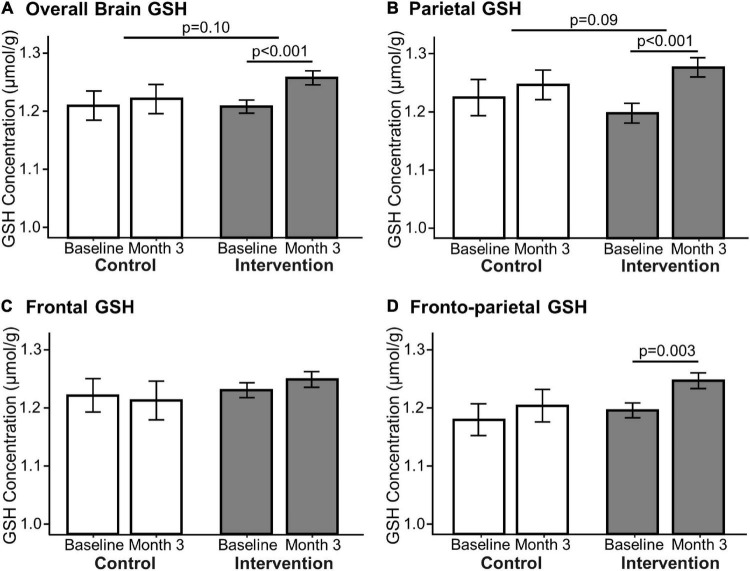
Effect of 3-month milk intervention on brain GSH concentrations. GSH concentrations in the overall brain (**A**: *p* < 0.001), parietal (**B**: *p* < 0.001), and fronto-parietal (**D:** *p* = 0.003) regions increased within the intervention group from baseline to the end of the study determined by paired *t*-test. There was no change in GSH concentration in the frontal region **(C)** nor in the control group between baseline and Month 3. Linear mixed models comparing changes in GSH concentration between the two groups indicated that the intervention group had a trending increase in overall (*p* = 0.10) and parietal (*p* = 0.09) GSH concentrations compared to the control group.

Brain GSH CSI data were processed using in-house software written in MATLAB (Mathworks, Framingham, MA, United States) for spatial reconstruction and post-processing including spatial alignment of CSI data acquired at baseline and 3-month follow-up scans. Three-dimensional T1-weighted MRI data were processed using SPM12 (UCL, London, United Kingdom). Spectral fitting of brain GSH and creatine signals was performed using in-house software written in IDL (RSI, Boulder, CO, United States) as described previously (1). Brain GSH concentrations were determined using an internal reference method utilizing the simultaneously acquired creatine CSI signals and given as μmol/g tissue. Creatine signals were quantified based on the unsuppressed water CSI signals. Brain GSH concentrations were then calculated from the central 7.5 × 7.5 × 2.5 cm^3^ region of the GSH CSI data. The total brain area for GSH quantification spans portions of the frontal and parietal regions and is labeled as “overall” (Figure 4). Brain GSH concentrations were obtained from the anterior one-third (2.5 × 7.5 cm^2^), the middle one-third (2.5 × 7.5 cm^2^), and the posterior one-third (2.5 × 7.5 cm^2^), and labeled as frontal, fronto-parietal and parietal, respectively.

### Statistical analysis

Descriptive statistics were presented as mean ± SD for all continuous variables and frequencies (relative frequency) for all categorical variables. Statistical analyses were assessed using data from all randomized participants who completed the study (*n* = 66). Independent samples *t*-tests and Pearson chi-square tests were used to compare characteristics between the intervention and control groups. We used paired sample *t*-tests to assess for within-group changes in dietary intake and serum cysteine levels. Based on our primary hypothesis that the 3-month milk intervention would increase brain GSH concentrations, this study was designed to detect a within-group change in brain GSH concentrations in the intervention group. Due to this and the uneven randomization scheme, we compared differences between pre- and post-intervention brain GSH concentrations within both the control and intervention groups using paired-sample *t*-tests. Although this study was not designed to determine between-group differences in brain GSH changes due to unequal randomization and a small sample size of the control group, we compared changes in brain GSH between the control and intervention groups by constructing linear mixed models of GSH change as a function of the interaction term of the group by time as a continuous variable and including subject as a random effect. To assess the influence of covariates on changes in brain GSH concentrations, we constructed linear mixed models that further adjusted for the fixed effects of age and sex. We determined Cohen’s d effect sizes from paired *t*-tests, partial eta-squared (η_p_^2^) effect sizes from LMMs, and *post-hoc* power for all analyses.

As secondary analyses to test the relationship between milk consumption and change in brain GSH, we used linear mixed models with brain GSH as a function of reported milk intake on 7DFRs, including a random effect for subject ID to account for repeated brain GSH measurement and individual variability in GSH concentrations. We also used Pearson’s correlation to test the correlation between reported changes in milk intake with changes in brain GSH concentrations from baseline to 3 months. All reported descriptive variables were assessed for normality with distribution histograms and statistical models were assessed for model assumptions using residual analyses (i.e., residual histograms and quantile-quantile plots). Statistical analyses were performed using R software (v. 3.6.1; R Foundation, Vienna, Austria). Statistical tests were two-tailed and significance was set at *p* < 0.05.

## Results

### Participants

From April 2017 to September 2019, a total of 148 individuals were assessed for study eligibility. Participant recruitment and flow are presented in Figure 1. We obtained consent from 80 participants (age 60–89 years) who enrolled in this study, of which 73 participants were randomized to either the intervention (*n* = 53) or control (*n* = 20) group. A total of 66 participants (*n* = 49 intervention and *n* = 17 controls) completed the study, defined by successful completion of brain GSH scans at baseline and Month 3. There were no demographic differences in participant characteristics between the intervention and control groups (Table 1). There were also no characteristic differences between participants who completed the study and participants who withdrew.

**TABLE 1:** Participant demographics*.

	Control (*n* = 17)	Intervention (*n* = 49)	*p*
Sex			0.54
Female, n (%)	11 (64.7%)	36 (73.5%)	
Male, n (%)	6 (35.3%)	13 (26.5%)	
Age, year	69.8 ± 6.0	69.0 ± 6.8	0.61
Race			0.79
Non-hispanic white	16 (94.1%)	45 (91.9%)	
Non-hispanic black	1 (5.9%)	1 (2.0%)	
Hispanic/latino	0 (0.0%)	2 (4.1%)	
Non-hispanic Asian	0 (0.0%)	1 (2.0%)	
Activity level			0.93
Inactive	1 (5.9%)	4 (8.3%)	
Light intensity	1 (5.9%)	6 (12.5%)	
Moderate intensity	6 (35.3%)	18 (37.5%)	
Hard intensity	4 (23.5%)	11 (22.9%)	
Very hard intensity	5 (29.4%)	9 (18.8%)	
Baseline BMI, kg/m^2^	25.4 ± 3.7	26.2 ± 3.9	0.58
Baseline waist circumference, cm	90.6 ± 9.8	91.1 ± 10.5	0.85

All values are shown in Mean ± SD.

*Group differences for continuous variables were assessed using two-sample *t*-tests.

Proportional differences among groups were assessed using Pearson’s chi-square.

### Dietary intake

Dietary intake from 7DFRs at baseline and the end of the study (Month 3) was converted into the intake of nutrients and food groups using NDSR software and is presented in Table 2 and Supplementary Table 1. At baseline, there were no group differences in dietary intake. The control group had similar milk intake at baseline and Month 3 timepoints (0.4 ± 0.4 cups/day for both; milk intake range at baseline: 0.0 – 1.1 cups/day and at Month 3: 0.0 – 1.3 cups/day). The intervention group had high compliance by increasing their 7DFR-reported milk intake from 0.2 ± 0.3 cups/day (range: 0.0 – 1.0 cups/day) to 3.0 ± 0.6 cups/day (*p* < 0.001). The intervention group showed increased intake of several milk-related nutrients (Table 2), which were higher than the intake in the control group at the end of the study. HEI scores for overall diet quality were similar within and between groups for both timepoints (baseline and Month 3).

**TABLE 2:** Differences in dietary intake by group*.

	Control (*n* = 17)			Intervention (*n* = 48)^†^		
	Baseline	Month 3	*P* ^#^	Baseline	Month 3	*p*
Energy, kcal	1760 ± 670 3	1770 ± 650	0.97	1700 ± 530	1770 ± 440	0.45
Total fat, g	77.7 ± 37.9	75.7 ± 30.6	0.87	71.7 ± 25.6	69.6 ± 22.3	0.67
Total Carbohydrate, g	192.3 ± 70.0	196.5 ± 69.9	0.86	191.3 ± 66.9	198.5 ± 56.7	0.57
Total protein, g	77.4 ± 31.9	71.7 ± 30.7	0.61	69.5 ± 21.3	85.1 ± 19.8	<0.001
HEI-2015	68.6 ± 14.2	69.9 ± 16.2	0.84	67.0 ± 12.0	66.7 ± 11.1	0.91
Total dairy	1.0 ± 0.5	0.9 ± 0.4	0.51	0.8 ± 0.4	3.5 ± 0.7	<0.001
Milk, cups	0.4 ± 0.4	0.4 ± 0.4	0.92	0.2 ± 0.3	3.0 ± 0.6	<0.001
Cheese, cups	0.5 ± 0.4	0.4 ± 0.3	0.33	0.5 ± 0.3	0.5 ± 0.3	0.41
Yogurt, cups	0.1 ± 0.1	0.1 ± 0.2	0.84	0.1 ± 0.2	<0.1 ± 0.1	0.13
**Micronutrients**						
Vitamin D, mcg	6.9 ± 6.3	5.9 ± 6.0	0.65	4.7 ± 3.1	10.7 ± 3.7	<0.001
Riboflavin, mg	1.9 ± 0.7	1.8 ± 0.7	0.94	1.9 ± 1.0	2.9 ± 0.8	<0.001
Vitamin B-12, mcg	4.1 ± 2.3	4.0 ± 2.2	0.83	3.9 ± 3.0	6.6 ± 2.7	<0.001
Calcium, mg	798.1 ± 220.6	732.7 ± 232.5	0.41	725.1 ± 245.7	1417.0 ± 317.6	<0.001
Phosphorus, mg	1195.3 ± 514.9	1142.2 ± 451.7	0.75	1093.4 ± 331.5	1569.0 ± 336.7	<0.001

All values are shown in Mean ± SD.

*Dietary intake was collected using 7-day food records and converted to intake of nutrients and food groups using NDSR software.

^#^Within-group differences between Baseline and Month 3 values were assessed using paired sample *t*-tests. Between-group differences at the end timepoint (Month 3) were assessed using two-sample *t*-tests.

^†^The intervention group was missing 7-day food records from one participant at baseline and the end of the study (Month 3).

### Serum cysteine

Cysteine levels in the blood were similar between the control and intervention groups at baseline (control: 0.60 ± 0.08 mM, intervention: 0.64 ± 0.11 mM, *p* = 0.61). Serum cysteine levels were unchanged in both groups from baseline to the end of the study (control: 0.58 ± 0.12 mM, intervention: 0.63 ± 0.11 mM, *p* = 0.66 for a between-group change).

### Intervention effect on brain glutathione concentrations

For brain GSH measurements, the unique spectral pattern of GSH signals from the cysteine β-CH_2_ protons (GSH Cys-β) was consistently observed at ∼3 ppm in the brains of all participants. The consistent measurements of brain GSH are demonstrated by partial views of GSH CSI at baseline (Figure 3B, left) and Month 3 (Figure 3B, right) from the same subject in the intervention group. GSH quantification was performed using creatine as an internal reference. Creatine concentrations did not differ between time points (baseline and Month 3) in all brain regions (*p* > 0.14). Both the intervention and control groups had similar GSH concentrations in all three (frontal, parietal and fronto-parietal) brain regions and the overall brain concentration at baseline (*p*-value range: 0.45 – 0.96).

The primary outcomes of this study are presented in Figure 4. GSH concentrations at baseline (1.23 ± 0.28 μmol/g) were comparable to our previous report in low to moderate dairy-consuming older adults (1.21 ± 0.09 μmol/g) (1). The intervention group demonstrated an increase of “overall” GSH concentration by 4.6 ± 8.7% (*p* < 0.001, Cohen’s d = 0.51, power = 0.94), an increase of parietal GSH by 7.4 ± 11.7% (*p* < 0.001, Cohen’s d = 0.62, power = 0.99), and an increase of fronto-parietal GSH by 4.7 ± 9.8% (*p* = 0.003, Cohen’s d = 0.45, power = 0.87) (Figure 4). The intervention group had no change in GSH concentrations in the frontal region. Brain GSH concentrations in all brain regions in the control group were unchanged from baseline to the end of the study (Month 3).

Although the study was not designed to adequately test for between-group differences in change, we explored differences in between-group effects on GSH concentration using linear mixed models. The intervention group had a trending increase in overall (4.6 ± 8.7% vs. 1.0 ± 4.3%, *p* = 0.10, η_p_^2^ = 0.03, power = 0.32) and parietal (7.4 ± 11.7% vs. 2.2 ± 8.8%, *p* = 0.09, η_p_^2^ = 0.03, power = 0.38) GSH relative to the control group, but not in the frontoparietal (*p* = 0.24) or frontal regions (*p* = 0.47). The results of the linear mixed models for between-group changes are also presented in Figure 4. Age and sex did not influence the between-group intervention effect.

### Secondary correlation analyses with longitudinal changes in brain glutathione

**FIGURE 5:**
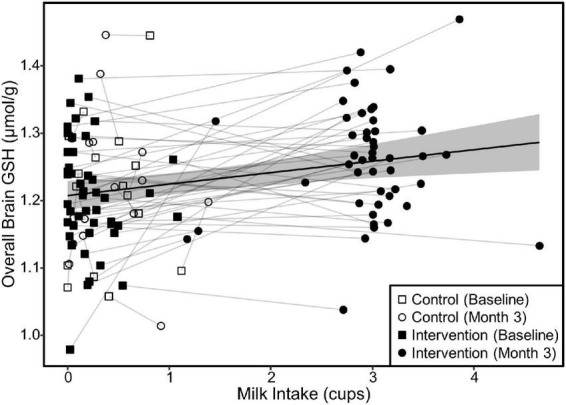
Relationship between milk intake and overall brain GSH concentration in both groups. The scatterplot demonstrates the relationship (β = 0.02, *p* < 0.001) between milk intake and GSH concentrations determined by a linear mixed model of overall brain GSH concentration as a function of milk intake including the random effect of subject ID to account for repeated measures and individual variance in GSH concentrations. Relationships were similar for the parietal and fronto-parietal regions (β = 0.02 and *p* < 0.001 for all). The shaded area represents the 95% confidence interval for the fixed effect of milk intake.

Our linear mixed models testing for the relationship of the fixed effect of milk intake on brain GSH concentrations accounting for a subject as a random effect indicated that increased milk intake was related to increased GSH concentrations overall (β = 0.02, *p* < 0.001), parietal (β = 0.03, *p* < 0.001), and frontoparietal regions (β = 0.02, *p* < 0.001). The fixed effect of milk intake is illustrated in Figure 5.

Serum cysteine levels were not correlated with brain GSH concentration at any timepoint, nor were changes in serum cysteine related to changes in brain GSH concentrations.

### Influence of baseline glutathione concentrations on intervention response

**FIGURE 6:**
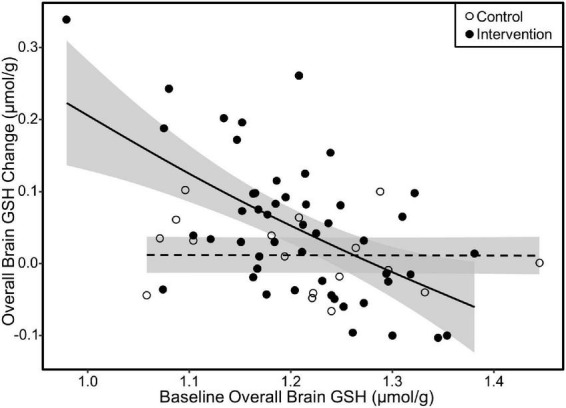
Scatterplot of change in overall GSH as a function of baseline overall GSH concentration. The fit lines represent regression estimated means for the milk intervention (solid line) and control (dashed line) groups. Response to the milk intervention was highest in those with lower baseline GSH concentrations with attenuating response as baseline GSH approached 1.4 μmol/g. There was no difference in GSH change due to baseline GSH in the control group. Similar relationships were also observed in the parietal and fronto-parietal regions. Shaded areas are 95% confidence intervals.

We investigated the influence of baseline GSH concentrations on intervention response by plotting the change in overall, parietal, and fronto-parietal GSH as a function of their respective baseline GSH concentrations. All three of these regions exhibited similar relationships with greater GSH increases by those with lower GSH concentrations at baseline in the intervention group. Irrespective of baseline GSH concentrations, individuals in the control group did not increase GSH levels. The plot results for overall brain GSH are presented in Figure 6. We subsequently tested the linear relationship between baseline GSH and GSH change among individuals in the intervention only. The increase in GSH (i.e., the intervention response) was attenuated by 0.07 μmol/g overall, 0.06 μmol/g in the parietal region, and 0.07 in the fronto-parietal region for every 0.1 μmol/g higher baseline GSH concentration (*p* < 0.001 for all regions).

We further tested whether the biological factors of sex or age influenced baseline GSH concentrations. There were no differences in overall or regional GSH between males and females (*p*-value range: 0.69 – 0.95), yet each additional year in age at baseline was related to 0.003 μmol/g lower overall (*p* < 0.05) and 0.005 μmol/g lower parietal (*p* = 0.03) GSH concentrations at baseline. Age was not related to baseline fronto-parietal (*p* = 0.17) nor frontal (*p* = 0.40) GSH concentrations.

## Discussion

This study demonstrates that increasing milk intake to the recommended 3 cups/day in older adults with habitual low intake raised brain GSH concentration over 3 months. This randomized, controlled clinical trial supports our previous finding that higher milk intake was cross-sectionally correlated with higher brain GSH concentrations in older adults, and extends this finding by suggesting the potential for a causal relationship. The effect of increased milk intake on changes in brain GSH concentrations was not identical in all measured brain regions with the most pronounced increases in the parietal region, indicating regional heterogeneity of the cerebral antioxidant system.

The milk intervention group had a 4.6% increase in overall brain GSH concentrations and a 7.4% increase in the parietal region. The previous findings of our lab (32) and others (33) have demonstrated that brain GSH concentration in older adults is significantly lower than in younger adults by over 10%. This result is significant and clinically meaningful because it demonstrates that age-related reduction of brain GSH could be restored by increasing the dietary intake of nutrients necessary for GSH synthesis (34). To date, aside from the milk intervention from the current study, few interventions have been demonstrated to increase brain GSH concentrations. For example, a small study with N-acetylcysteine (NAC) infusion reported acute increases in brain GSH concentrations in three healthy older adults (35), while oral doses of NAC have been ineffective (36). In this regard, modulating brain GSH concentrations *via* dietary intake could be a simple and safe option for the general public without any potential drug interactions or adverse effects. Our results are particularly encouraging because milk is widely available in grocery stores as a common food in the US and most other countries and is included as a healthy beverage option in several federal nutrition assistance programs (37).

Providing further context to the findings of our study, we explored biological factors that may have influenced the impact of the milk intervention. GSH concentrations in the overall, parietal, and fronto-parietal regions increased in the milk intervention group. However, there was variability in GSH changes in response to the milk intervention. We observed that individuals with lower overall and parietal GSH concentrations at baseline had significantly larger GSH increases in response to the milk intervention, whereas those in the control group did not show any changes in GSH concentrations, irrespective of GSH concentrations at baseline. These findings have important implications for designing future clinical trials to assess the efficacy of dairy and other nutrition interventions in improving brain antioxidant status. These dietary interventions may be most effective in those with lower antioxidant status due to higher age or increased oxidative stress. As the field aims to better cater precise nutrition interventions to specific populations, these phenotypic characterizations will be important for screening in future studies.

In addition, among the cohort of our study participants, individuals of higher age had lower GSH concentrations in the overall and parietal regions at baseline. These results are consistent with our previous and other findings regarding lower brain GSH in aging and Alzheimer’s disease (32, 33, 38, 39). However, some studies report conflicting findings, i.e., higher (40, 41) or no changes (42) in brain GSH concentrations. This seeming discrepancy could be due to differences in measurement methods (e.g., MR sequences and quantification methods) and sampled brain regions, thus warrants further investigation.

Our results also demonstrate that it is possible for older adults who are low milk consumers to consume 3 cups of milk daily without increasing their energy intake. Our intervention education consisted of strategies to incorporate 3 cups of milk into daily consumption, yet did not include strategies to offset caloric intake due to the addition of milk into the diet. It is unclear whether caloric intake was unaffected by the milk intervention due to a conscious effort by participants to offset caloric take or a physiologic satiety mechanism. As 3 cups of milk are consistent with Dietary Guidelines for Americans (see text footnote 1), our data suggest that older adults can add 1% milk to their daily diet without affecting energy balance. As a result, the intervention group increased its intake of milk-related nutrients including protein, vitamin D, riboflavin, vitamin B12, calcium, and phosphorus (Table 2). Our intervention only included increasing daily milk intake from <1 cup/day to 3 cups/day, and no changes were observed in other food groups or diet quality. Thus, it is unlikely that the results of this study can be contributed to any factor other than increased milk intake.

The results from this randomized clinical trial extend our prior findings in an observational study demonstrating higher brain GSH concentration in those who consumed higher dairy consumption; yet, the mechanisms by which milk influences brain GSH concentrations are still unknown. In addition to riboflavin and calcium that support the maintenance of GSH, milk is a source of the amino acids, glycine, glutamate, and cysteine that are required for GSH synthesis. The whey fraction of milk may be beneficial as it is rich in cysteine (15), the rate-limiting amino acid for GSH synthesis. Because the nutrient database used in this study did not provide an estimate for cysteine intake, we were unable to analyze the relationship between dietary cysteine and change in brain GSH. Thus, we aimed to investigate the role of cysteine availability in brain GSH changes by measuring fasted serum cysteine; however, we did not find any changes in serum cysteine after the intervention. This is likely because cysteine is rapidly metabolized in the blood, and more sensitive methods would be required to detect any differences after dietary interventions. Understanding the potential mechanisms by which milk consumption influences brain GSH concentrations requires further investigation in future studies.

Major strengths of the study include the randomized controlled design with blinding of study personnel and the rigor of the study methods where milk was provided to participants weekly with milk measuring containers, daily self-adherence tracking by participants, and rigorous weekly monitoring of study adherence of participants by study staff. Additionally, we monitored total diet intake to control for potential other dietary factors that could influence the outcomes. Another strength is a direct measurement of brain GSH using the novel multiple quantum CSI of GSH to measure the effect of dietary milk intervention on the living brain tissue and to assess regional variabilities of GSH changes.

While this study provides evidence that increasing milk consumption may increase brain GSH concentrations, some limitations should be noted. The small sample size of the control group due to our unequal randomization resulted in wide variability in the control group, ultimately limiting our ability to assess for true causal relationships by adequately testing for between-group differences. This study was conducted with a specific group of older adults with low dairy consumption. Our results showed that increasing milk intake from low to the recommended levels can raise brain GSH concentrations. However, it is unknown whether the same effect would be observed in older adults with moderate milk intake or whether consuming more than 3 cups/day would provide added benefit. Also, because we used one type of milk, i.e., 1% milk, in this intervention, it is unclear whether we can observe the same findings from milk with different levels of fat contents. Lastly, the widely used serum cysteine assay method we chose to analyze total serum cysteine using a fluorometric assay kit may not have been sensitive to subtle cysteine changes in the blood after increased milk intake.

Future research is warranted to determine the dose-response to different amounts, whether fat content and species of milk have a differential effect on brain GSH concentrations, and the potential benefits of high GSH in the brain (i.e., better cognitive function).

## Conclusion

We demonstrated that a 3-month milk intervention of the recommended 3 cups/day among healthy older adults with habitual low milk intake effectively increased brain GSH concentrations. To our knowledge, this is the first randomized controlled trial to demonstrate increases in brain GSH concentrations that coincide with a dietary intervention with milk. This suggests that milk is an important dietary source that enhances brain GSH and its consumption could be a strategy that can be easily accessible to the public for strengthening cerebral antioxidant defenses to promote brain health in the aging population.

## Data availability statement

The raw data supporting the conclusions of this article will be made available by the authors, without undue reservation.

## Ethics statement

The studies involving human participants were reviewed and approved by the Institutional Review Board at the University of Kansas Medical Center. The patients/participants provided their written informed consent to participate in this study.

## Author contributions

I-YC, PL, and DS designed the research. I-YC, MT, PL, SA, and KS performed the research. I-YC, PL, and PA analyzed the MR data. MT, SA, and DS analyzed the diet data. MB and JH-R performed the blood cysteine analysis. MT performed the statistical analysis. I-YC, PL, MT, and DS wrote the manuscript. All authors contributed to the article and approved the submitted version.

## Footnotes

1
www.dietaryguidelines.gov
