# Supplementary material for: Milk intake enhances cerebral antioxidant (glutathione) concentration in older adults: A randomized controlled intervention study
Source: Front Nutr. 2022 Aug 15;9:811650. doi: 10.3389/fnut.2022.811650 (PMC9421260; doi:10.3389/fnut.2022.811650)
Supplement: Supplementary file 1 [file Table_1.DOCX]

Supplementary Material

# Supplemental Table 1. Comprehensive Dietary Intake

|  | **Control (n=17)** | | |  | **Intervention (n=48)** | | |
| --- | --- | --- | --- | --- | --- | --- | --- |
|  | **Baseline** | **Month 3** | **p** |  | **Baseline** | **Month 3** | **p** |
| Energy (kcal) | 1760 ± 670 | 1770 ± 650 | 0.97 |  | 1700 ± 530 | 1770 ± 440 | 0.45 |
| Total Fat (g) | 77.7 ± 37.9 | 75.7 ± 30.6 | 0.87 |  | 71.7 ± 25.6 | 69.6 ± 22.3 | 0.67 |
| Cholesterol (mg) | 250.2 ± 151.1 | 273.5 ± 168.8 | 0.68 |  | 255.2 ± 131.3 | 273.1 ± 127.1 | 0.50 |
| Saturated Fatty Acids (g) | 23.2 ± 9.5 | 22.6 ± 9.6 | 0.87 |  | 22.6 ± 8.8 | 25.0 ± 8.2 | 0.17 |
| Monounsaturated Fatty Acids (g) | 28.1 ± 16.3 | 27.1 ± 12.8 | 0.85 |  | 27.2 ± 10.9 | 25.0 ± 8.7 | 0.30 |
| Polyunsaturated Fatty Acids (g) | 19.8 ± 12.2 | 19.7 ± 10.5 | 0.99 |  | 16.1 ± 6.3 | 13.9 ± 5.2 | 0.07 |
| Trans-Fatty Acids (g) | 1.6 ± 0.7 | 1.6 ± 0.8 | 0.83 |  | 1.7 ± 1.0 | 2.1 ± 1.0 | 0.04 |
| Omega-3 Fatty Acids (g) | 2.5 ± 2.2 | 2.6 ± 1.9 | 0.91 |  | 1.8 ± 0.8 | 1.4 ± 0.5 | 0.01 |
| Total Carbohydrate (g) | 192.3 ± 70.0 | 196.5 ± 69.9 | 0.86 |  | 191.3 ± 66.9 | 198.5 ± 56.7 | 0.57 |
| Total Sugars (g) | 78.7 ± 37.9 | 80.6 ± 40.5 | 0.89 |  | 78.5 ± 34.8 | 97.4 ± 31.7 | 0.01 |
| Total Dietary Fiber (g) | 23.7 ± 12.8 | 22.2 ± 10.1 | 0.71 |  | 21.0 ± 8.0 | 18.0 ± 6.0 | 0.04 |
| Soluble Dietary Fiber (g) | 6.4 ± 2.5 | 6.2 ± 2.4 | 0.80 |  | 6.7 ± 2.3 | 5.9 ± 1.9 | 0.10 |
| Insoluble Dietary Fiber (g) | 17.1 ± 10.6 | 15.9 ± 8.0 | 0.73 |  | 14.2 ± 6.1 | 12.0 ± 4.8 | 0.05 |
| Total Protein (g) | 77.4 ± 31.9 | 71.7 ± 30.7 | 0.61 |  | 69.5 ± 21.3 | 85.1 ± 19.8 | <0.001 |
| Animal Protein (g) | 50.5 ± 21.8 | 45.7 ± 24.6 | 0.56 |  | 43.7 ± 16.7 | 62.8 ± 18.6 | <0.001 |
| **HEI-2015** | 68.6 ± 14.2 | 69.9 ± 16.2 | 0.84 |  | 67.0 ± 12.0 | 66.7 ± 11.1 | 0.91 |
| Total Fruit | 3.3 ± 1.7 | 3.5 ± 1.6 | 0.77 |  | 3.0 ± 1.6 | 2.3 ± 1.4 | 0.03 |
| Whole Fruit | 4.0 ± 1.7 | 4.2 ± 1.2 | 0.64 |  | 3.8 ± 1.6 | 3.4 ± 1.7 | 0.23 |
| Total Vegetables | 4.2 ± 1.1 | 3.8 ± 1.1 | 0.30 |  | 3.9 ± 1.3 | 3.6 ± 1.2 | 0.29 |
| Greens and Beans | 3.6 ± 1.8 | 4.0 ± 1.3 | 0.46 |  | 3.4 ± 1.8 | 3.1 ± 1.7 | 0.31 |
| Whole Grains | 4.5 ± 3.1 | 5.8 ± 3.1 | 0.24 |  | 6.3 ± 3.0 | 5.5 ± 3.3 | 0.23 |
| Dairy | 6.1 ± 2.4 | 5.3 ± 2.0 | 0.32 |  | 4.8 ± 2.2 | 10.0 ± 0.1 | <0.001 |
| Total Protein Foods | 4.8 ± 0.4 | 4.8 ± 0.9 | 0.81 |  | 4.9 ± 0.4 | 4.7 ± 0.7 | 0.07 |
| Seafood and Plant Proteins | 4.4 ± 1.3 | 4.6 ± 1.3 | 0.80 |  | 4.5 ± 1.1 | 3.9 ± 1.5 | 0.04 |
| Fatty Acids | 5.6 ± 3.3 | 5.9 ± 3.3 | 0.83 |  | 5.5 ± 2.7 | 3.0 ± 2.4 | <0.001 |
| Refined Grains | 8.0 ± 2.8 | 7.6 ± 3.1 | 0.74 |  | 8.0 ± 2.5 | 8.6 ± 1.8 | 0.19 |
| Sodium | 5.4 ± 3.2 | 5.5 ± 2.7 | 0.95 |  | 5.0 ± 3.0 | 4.9 ± 3.1 | 0.90 |
| Added Sugars | 9.0 ± 1.4 | 9.0 ± 1.3 | 0.94 |  | 8.3 ± 1.8 | 9.0 ± 1.4 | 0.03 |
| Saturated Fats | 5.6 ± 3.1 | 5.7 ± 3.4 | 0.93 |  | 5.6 ± 2.8 | 4.7 ± 2.7 | 0.11 |
| **Amino Acids** |  |  |  |  |  |  |  |
| Tryptophan (g) | 0.9 ± 0.4 | 0.8 ± 0.4 | 0.56 |  | 0.8 ± 0.3 | 1.0 ± 0.3 | <0.001 |
| Threonine (g) | 3.0 ± 1.3 | 2.8 ± 1.3 | 0.60 |  | 2.7 ± 0.8 | 3.4 ± 0.8 | <0.001 |
| Isoleucine (g) | 3.4 ± 1.4 | 3.1 ± 1.4 | 0.58 |  | 3.0 ± 1.0 | 3.9 ± 1.0 | <0.001 |
| Leucine (g) | 6.0 ± 2.4 | 5.5 ± 2.4 | 0.55 |  | 5.3 ± 1.7 | 6.9 ± 1.6 | <0.001 |
| Lysine (g) | 5.2 ± 2.2 | 4.7 ± 2.3 | 0.55 |  | 4.5 ± 1.5 | 5.9 ± 1.5 | <0.001 |
| Methionine (g) | 1.7 ± 0.8 | 1.6 ± 0.7 | 0.59 |  | 1.5 ± 0.5 | 2.0 ± 0.5 | <0.001 |
| Cystine (g) | 1.0 ± 0.4 | 1.0 ± 0.4 | 0.70 |  | 0.9 ± 0.3 | 1.0 ± 0.3 | 0.41 |
| Phenylalanine (g) | 3.4 ± 1.4 | 3.1 ± 1.3 | 0.58 |  | 3.0 ± 0.9 | 3.9 ± 0.9 | <0.001 |
| Tyrosine (g) | 2.6 ± 1.0 | 2.4 ± 1.0 | 0.53 |  | 2.3 ± 0.7 | 3.2 ± 0.7 | <0.001 |
| Valine (g) | 3.9 ± 1.6 | 3.6 ± 1.5 | 0.53 |  | 3.4 ± 1.1 | 4.6 ± 1.1 | <0.001 |
| Arginine (g) | 4.4 ± 2.2 | 4.1 ± 1.9 | 0.66 |  | 3.9 ± 1.3 | 4.1 ± 1.0 | 0.45 |
| Histidine (g) | 2.1 ± 0.9 | 1.9 ± 0.8 | 0.52 |  | 1.9 ± 0.6 | 2.4 ± 0.6 | <0.001 |
| Alanine (g) | 3.7 ± 1.7 | 3.4 ± 1.6 | 0.60 |  | 3.3 ± 1.1 | 3.8 ± 1.0 | 0.03 |
| Aspartic Acid (g) | 7.1 ± 3.1 | 6.5 ± 2.9 | 0.56 |  | 6.3 ± 2.1 | 7.5 ± 1.8 | 0.002 |
| Glutamic Acid (g) | 14.4 ± 5.7 | 13.2 ± 5.0 | 0.49 |  | 13.1 ± 3.8 | 16.9 ± 3.7 | <0.001 |
| Glycine (g) | 3.2 ± 1.5 | 3.0 ± 1.3 | 0.58 |  | 2.9 ± 1.0 | 3.1 ± 0.8 | 0.30 |
| Proline (g) | 4.6 ± 1.7 | 4.2 ± 1.5 | 0.51 |  | 4.2 ± 1.2 | 6.1 ± 1.3 | <0.001 |
| Serine (g) | 3.6 ± 1.6 | 3.3 ± 1.4 | 0.57 |  | 3.1 ± 1.0 | 4.1 ± 0.9 | <0.001 |

Note: All values are shown in Mean ± SD. Within-group differences between Baseline and Month 3 values were assessed using paired sample t-tests.
